# Characteristics of demersal fish community structure during summer hypoxia in the Pearl River Estuary, China

**DOI:** 10.1002/ece3.11722

**Published:** 2024-07-11

**Authors:** Han Lai, Sheng Bi, Huadong Yi, Haiyang Li, Xuchong Wei, Gongpei Wang, Dingli Guo, Xuange Liu, Jiahui Chen, Qiuxian Chen, Zhilun Zhang, Shuang Liu, Chenlei Huang, Li Lin, Guifeng Li

**Affiliations:** ^1^ State Key Laboratory of Biocontrol Southern Marine Science and Engineering Guangdong Laboratory (Zhuhai) Guangdong Provincial Observation and Research Station for Marine Ranching in Lingdingyang Bay School of Life Sciences Sun Yat‐Sen University Guangzhou China; ^2^ Southern Marine Science and Engineering Guangdong Laboratory (Guangzhou) Guangzhou China; ^3^ Bureau of Hydrology Pearl River Water Resources Commission of the Ministry of Water Resources Guangzhou China

**Keywords:** dissolved oxygen, environmental factors, fish community, hypoxia, Pearl River Estuary

## Abstract

In recent decades, hypoxic areas have rapidly expanded worldwide in estuaries and coastal zones. The Pearl River Estuary (PRE), one of China's largest estuaries, experiences frequent seasonal hypoxia due to intense human activities and eutrophication. However, the ecological effects of hypoxia in the PRE, particularly on fish communities, remain unclear. To explore these effects, we collected fish community and environmental data in July 2021 during the summer hypoxia development period. The results revealed that bottom‐layer dissolved oxygen (DO) in the PRE ranged from 0.08 to 5.71 mg/L, with extensive hypoxic zones (DO ≤ 2 mg/L) observed. Hypoxia has varied effects on fish community composition, distribution, species, and functional diversity in the PRE. A total of 104 fish species were collected in this study, with approximately 30 species (28.6%) exclusively found in hypoxic areas. Species responses to hypoxia varied: species such as *Sardinella zunasi*, *Coilia mystus*, and *Nuchequula nuchalis* were sensitive, while *Decapterus maruadsi*, *Siganus fuscescens*, and *Lagocephalus spadiceus* showed higher tolerance. Within the hypoxia area, dissolved oxygen was the main limiting factor for fish community diversity. Functional diversity (FDiv) decreased with higher dissolved oxygen levels, indicating a potential shift in the functional traits and ecological roles of fish species in response to changing oxygen conditions. Further analysis demonstrated that dissolved oxygen had a significantly stronger effect on fish community structure at hypoxic sites than in the whole PRE. Moreover, other environmental variables also had significant effects on the fish community structure and interacted with dissolved oxygen in the hypoxia area. These findings suggest that maintaining sufficient dissolved oxygen levels is essential for sustaining fish communities and ecosystem health in the PRE. This study provides novel insights into the effects of hypoxia on fish communities in estuarine ecosystems and has significant implications for the ecological health and management of the PRE.

## Graphical abstract

Hypoxia affects fish community composition, distribution, diversity, and structure in the PRE. Dissolved oxygen was the main limiting factor for fish community diversity in the hypoxia area. Maintaining sufficient dissolved oxygen levels is essential for sustaining fish communities and ecosystem health in the PRE.

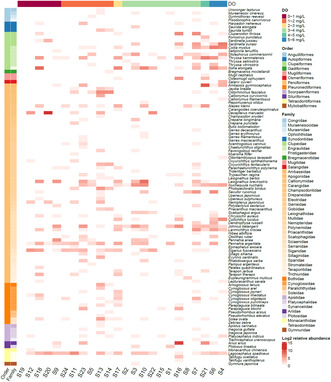

## 1 INTRODUCTION

Hypoxia refers to a condition in aquatic environments where dissolved oxygen (DO) levels decline to critically low thresholds, typically defined as below 2 mg/L, although there is no universal consensus on the exact threshold (Hofmann et al., 2011; Turner et al., 2005; Yin et al., 2004). In recent decades, hypoxia in estuarine and coastal waters has become a global environmental issue, with the frequency and severity of hypoxic events rapidly increasing (Diaz & Rosenberg, 2008). Since 1950, more than 500 locations in coastal waters worldwide have reported low oxygen (DO ≤ 2 mg/L) (Breitburg et al., 2018). The occurrence of hypoxia is usually the result of the interaction of various physical factors, such as water stratification, and biogeochemical processes, including eutrophication and respiratory oxygen consumption (Breitburg et al., 2018; Li et al., 2020).

The Pearl River Estuary (PRE) is one of the largest estuaries in China, characterized by a complex hydrodynamic system influenced by various interacting factors, including freshwater discharge, tidal mixing, and monsoon winds (Shen et al., 2022). The PRE supports a rich biodiversity of marine organisms and provides important ecosystem services such as fisheries, tourism, and transportation. Hypoxia was first observed in the PRE in 1985 (Lin et al., 2001). Since around 2000, there has been a significant expansion of summer hypoxic conditions at the bottom of the PRE, indicating a shift from a system with occasional, small‐scale hypoxic events (DO < 2 mg/L) to one experiencing seasonal, large‐scale hypoxia during summer (Hu et al., 2021). The occurrence of seasonal hypoxia in the PRE is primarily concentrated on the west and east shoals of the Lingdingyang Estuary and in the Modaomen Estuary (Cui et al., 2019). An analysis of intra‐annual variations in dissolved oxygen distribution in the PRE shows that, compared with the surface water, which maintains high dissolved oxygen concentrations year‐round, the bottom water exhibits significant seasonal fluctuations in dissolved oxygen levels. Hypoxia in the bottom water begins in May, peaks in August, and then gradually dissipates during the autumn and winter seasons until it disappears (Zhang et al., 2022).

Fish constitute a vital component of estuarine ecosystems and play a critical role in the food web. However, fish are also considered among the most sensitive marine organisms to hypoxia (Vaquer‐Sunyer & Duarte, 2008). Severe hypoxic conditions can lead to physiological damage, behavioral changes, reduced feeding, and habitat compression of non‐tolerant species, thus altering community structure (Keller et al., 2015; Koslow et al., 2011). Extensive research has been conducted on the fish community in the PRE, covering various aspects of their ecology. Traditional investigation methods have been used to analyze the composition and species diversity of the fish community, as well as the impacts of environmental changes on these communities (Zhou et al., 2019). Studies have also focused on the early life history of fish, their trophic structure, and food webs (Wang et al., 2023; Xu et al., 2022; Zhou et al., 2022). With the development of high‐throughput technology, environmental DNA (eDNA)‐based methods have recently been applied to assess fish diversity in the PRE (Cheang et al., 2020; Zou et al., 2020). Additionally, studies on the functional structures of fish communities in the estuary have gradually received attention (Kuang et al., 2021; Lai et al., 2022). Despite worsening dissolved oxygen conditions over the past three decades, leading to frequent hypoxia events, limited research has been conducted on the effects of hypoxia on the fish communities in the PRE. Understanding these effects is crucial for assessing the ecological consequences of hypoxia and developing effective management strategies to mitigate its impacts.

To illustrate the effects of summer hypoxia on demersal fish community structure in the PRE, we conducted a field survey to collect fish community and environmental data in July 2021 during the summer hypoxia development period. We analyzed the spatial distribution of hypoxia and its correlation with environmental variables and assessed the effects of hypoxia on fish community composition, species diversity, and functional diversity using various statistical methods. This study contributes to understanding the ecological impacts of hypoxia on fish communities in the PRE and provides valuable information for the management and conservation of the estuarine ecosystem.

## 2 METHODS

### 2.1 Study area and sampling design

**FIGURE 1:**
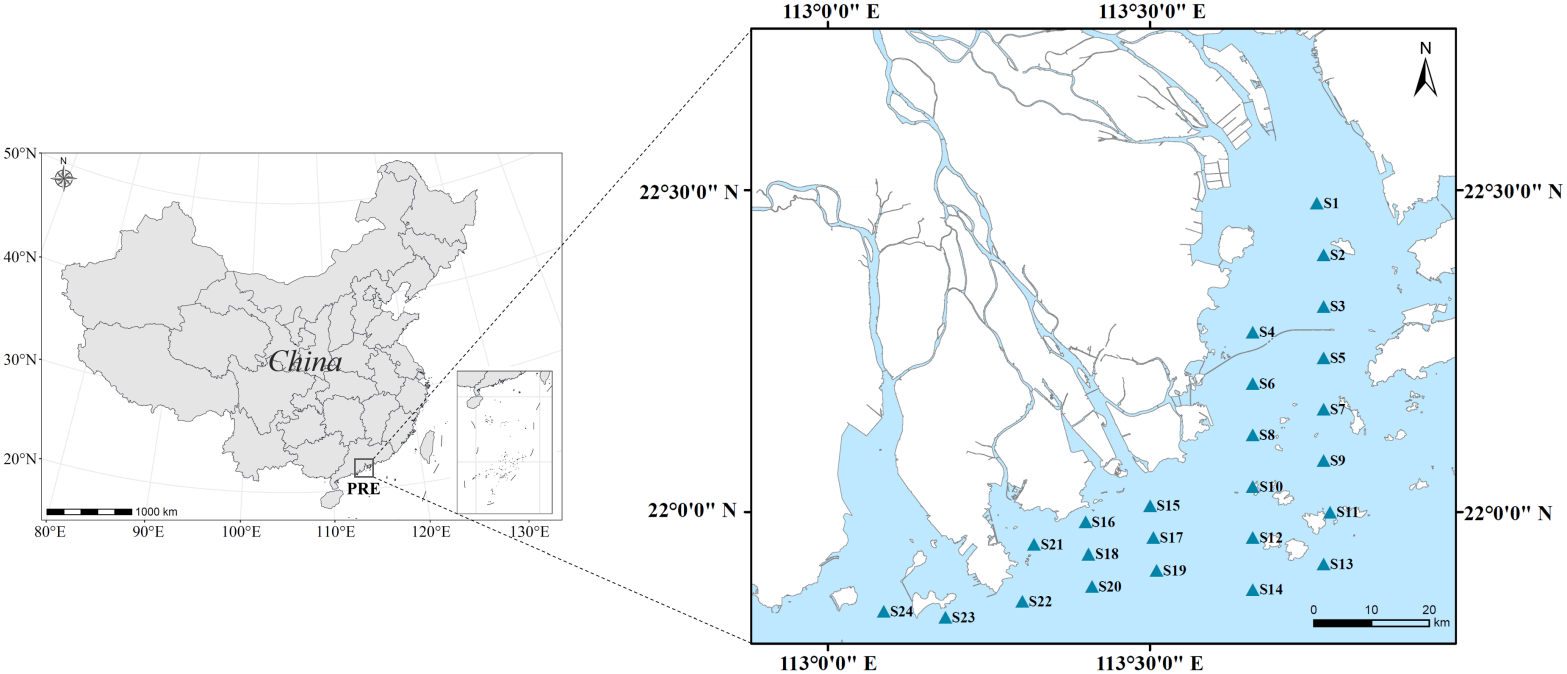
Map of sampling sites in the Pearl River Estuary.

The study was conducted in the Pearl River Estuary (PRE), located in southern China (Figure 1), which harbors abundant fishery resources and biodiversity (Xu et al., 2022). However, extensive human activities have led to a significant increase in nutrient emissions in the PRE, resulting in frequent and severe eutrophication and hypoxic events in recent decades. In this study, a total of 24 sampling sites in the estuary were selected, covering a wide range of dissolved oxygen concentrations, including areas with high frequency of occurrence of hypoxic events in the PRE. To investigate the effects of hypoxia on fish communities, we conducted our survey during a typical hypoxic period in the PRE (July 2021). This period coincided with the summer fishing moratorium, during which fishing activities and other disturbances were minimized, allowing for a more precise assessment of the effects of hypoxia on the fish communities in the PRE. We utilized a fishing vessel named “Yue Dongguan Yu 92096” (total tonnage: 127 tons, total power of main engine: 138.0 kW) for this investigation and obtained a special fishing license.

At each site, we employed two identical shrimp trawls (5.0 m long, 1.7 m opening diameter, 25 cm vertical opening height, and 25 mm mesh size) to collect fish samples. The trawls were deployed for 30 min at a speed of 4 knots, and all captured fish were identified, counted, and weighed onboard.

Environmental factors (temperature, salinity, dissolved oxygen, pH, and ammonium) were simultaneously measured at the surface and near the bottom of the water column using a calibrated Macro 900 multi‐parameter water quality analyzer. Water depth was measured using the BioSonics DT‐X echosounder. Chlorophyll‐a data were obtained from satellite data available on the Aqua/MODIS website (https://worldview.earthdata.nasa.gov/). Given the use of a bottom trawl survey, subsequent analysis primarily utilized environmental variables from the bottom layer.

### 2.2 Data analysis

To visualize the spatial distribution of water depth and environmental variables, including the distribution of hypoxia areas in the PRE, interpolation analysis was performed using ArcGIS 10.8 software. Relationships between various environmental variables were examined using the “ggpairs” function from the GGally package in R. To investigate the impact of hypoxia on fish species, we generated a relative abundance heatmap of fish species at different sites in the PRE using the “ComplexHeatmap” package in R (Gu, 2022). Additionally, we created a heatmap to explore the correlation between environmental variables and species.

We assessed the effects of hypoxia on fish community diversity by calculating species and functional diversity indices. Four species diversity indices were used: Shannon diversity index, Simpson diversity index (Gini‐Simpson index), Margalef richness index, and Pielou evenness index, calculated using PRIMER (version 7.0.10). The formulas for these indices are provided in Table S1. Functional diversity was assessed using five indices: functional richness (FRic) (Cornwell et al., 2006; Villeger et al., 2008), functional evenness (FEve) (Mason et al., 2005; Villeger et al., 2008), functional divergence (FDiv) (Villeger et al., 2008), functional dispersion (FDis) (Laliberte & Legendre, 2010), and Rao's quadratic entropy (RaoQ) (Botta‐Dukat, 2005; Mouchet et al., 2010), calculated using the “FD” package in R (Laliberte & Legendre, 2010). The functional diversity indices were calculated based on 10 traits (Table S2), including body form, position of mouth, swimming mode, length at maturity, age at maturity, life span, Estuary Use Functional Guild (EUFG), trophic guild, and trophic level. Trait data were primarily obtained from the FishBase database (www.fishbase.org) using the “rfishbase” package (Boettiger et al., 2012). EUFG data were sourced from https://www.dassh.ac.uk/doitool/data/1678 (Elliott et al., 2007; Harrison & Whitfield, 2021). Trophic guild data were classified by referencing the FishBase database, books, and related literature (Elliott et al., 2007). These traits provide a comprehensive description of the ecological characteristics of fish in estuarine environments and have been widely used to study the functional structure of fish communities (Lai et al., 2022).

Using the species and functional diversity indices, we employed generalized additive models (GAM) to examine relationships between diversity indices and dissolved oxygen levels. We analyzed overall diversity‐environment relationships in the PRE and specifically within the hypoxia area. Furthermore, to explore differences in fish community structure between hypoxia and normoxia areas, we conducted Non‐metric Multidimensional Scaling (NMDS) analysis based on Bray–Curtis dissimilarity matrices and assessed the significance of differences using permutational multivariate analysis of variance (PERMANOVA) analysis. GAMs were implemented using the “gam” function from the mgcv package in R, while NMDS was conducted using the “metaMDS” function from the vegan package in R. Additionally, the Mantel test, based on Spearman's correlation, was performed to further examine correlations between fish community structure and environmental factors in hypoxia areas, using the “vegan” package in R (Oksanen et al., 2022).

All statistical analyses were performed in R software version 4.3.0 (R Core Team, 2023).

## 3 RESULTS

### 3.1 Environmental characteristics and distribution of the hypoxia area

**FIGURE 2:**
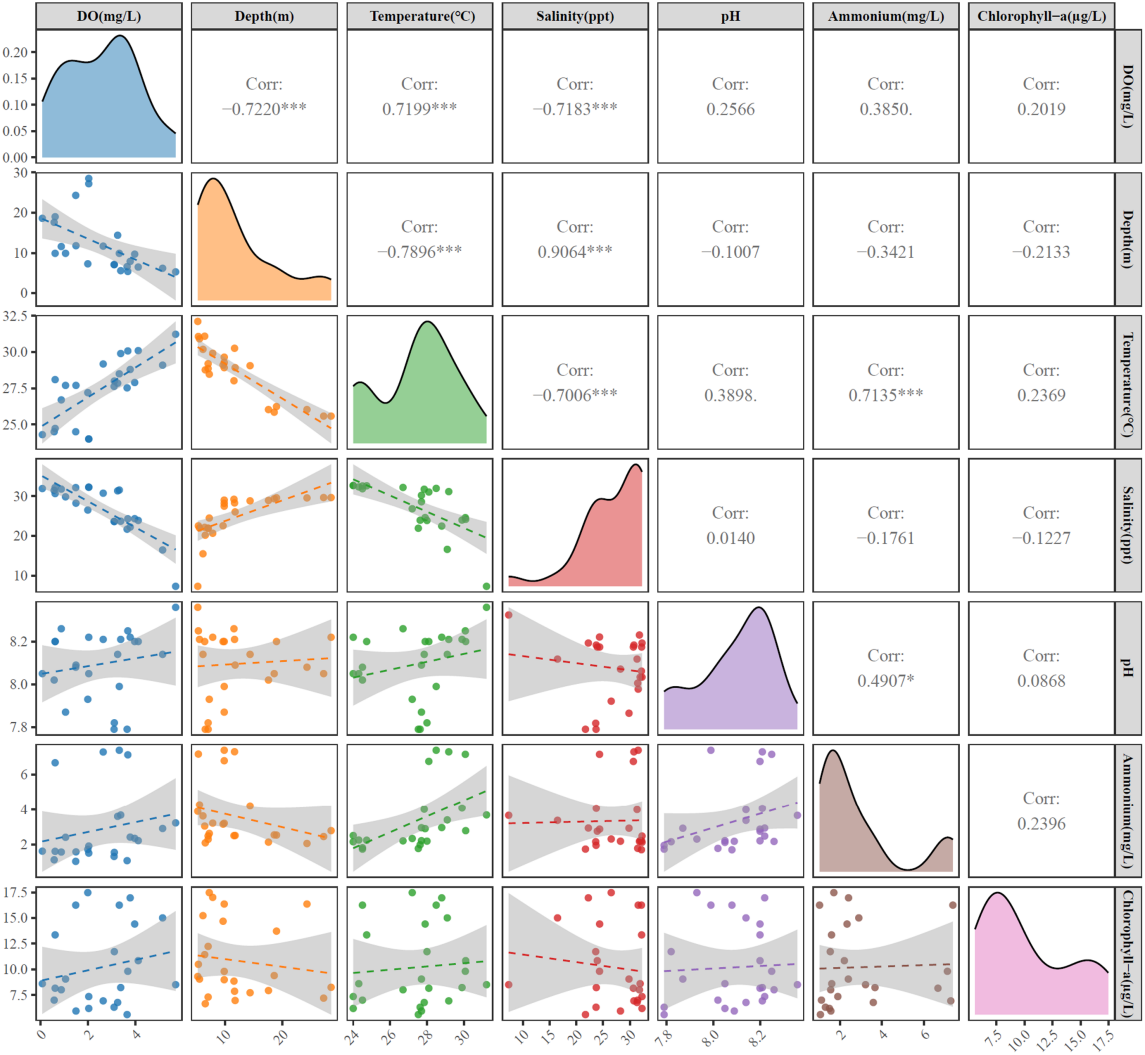
Correlation analysis of environmental variables in the Pearl River Estuary. (The lower panels show the relationships between environmental variables with linear trend lines. Diagonal panels display the density distributions of each variable. The upper panels represent Spearman's correlation coefficients (*R*), with significance levels indicated as follows: *p* < .05 (⁎), *p* < .01(⁎), and *p* < .001(⁎⁎⁎))

According to the spatial distribution of environmental variables at each sampling site (Figures S1 and S2) and the correlation analysis of bottom water variables (Figure 2), there are a consistent trend between temperature and dissolved oxygen in the bottom layer, with a significant positive correlation observed (*r* = .7199, *p* < .001). In contrast, salinity showed an inverse pattern and had a significant negative correlation with dissolved oxygen (*r* = −.7183, *p* < .001). In terms of depth distribution, the stations with lower bottom dissolved oxygen are mainly located in the deeper area outside the estuary. Additionally, sites with low bottom dissolved oxygen (e.g., S11, S12, S13, S14, S19, and S20) also exhibit low bottom temperatures, and there was a large difference between the surface and bottom‐layer temperatures, indicating that there might be a distinct thermocline that inhibited the vertical mixing and exchange of dissolved oxygen in the water column, which was also one of the important reasons for the formation of hypoxia areas. Although the concentration of bottom‐layer dissolved oxygen in the sampling sites of the PRE was significantly low, the surface‐layer dissolved oxygen was consistently high, and hypoxic conditions were not observed. The average dissolved oxygen concentration in the surface layer was 7.75 mg/L, with a minimum concentration of 6.15 mg/L.

**FIGURE 3:**
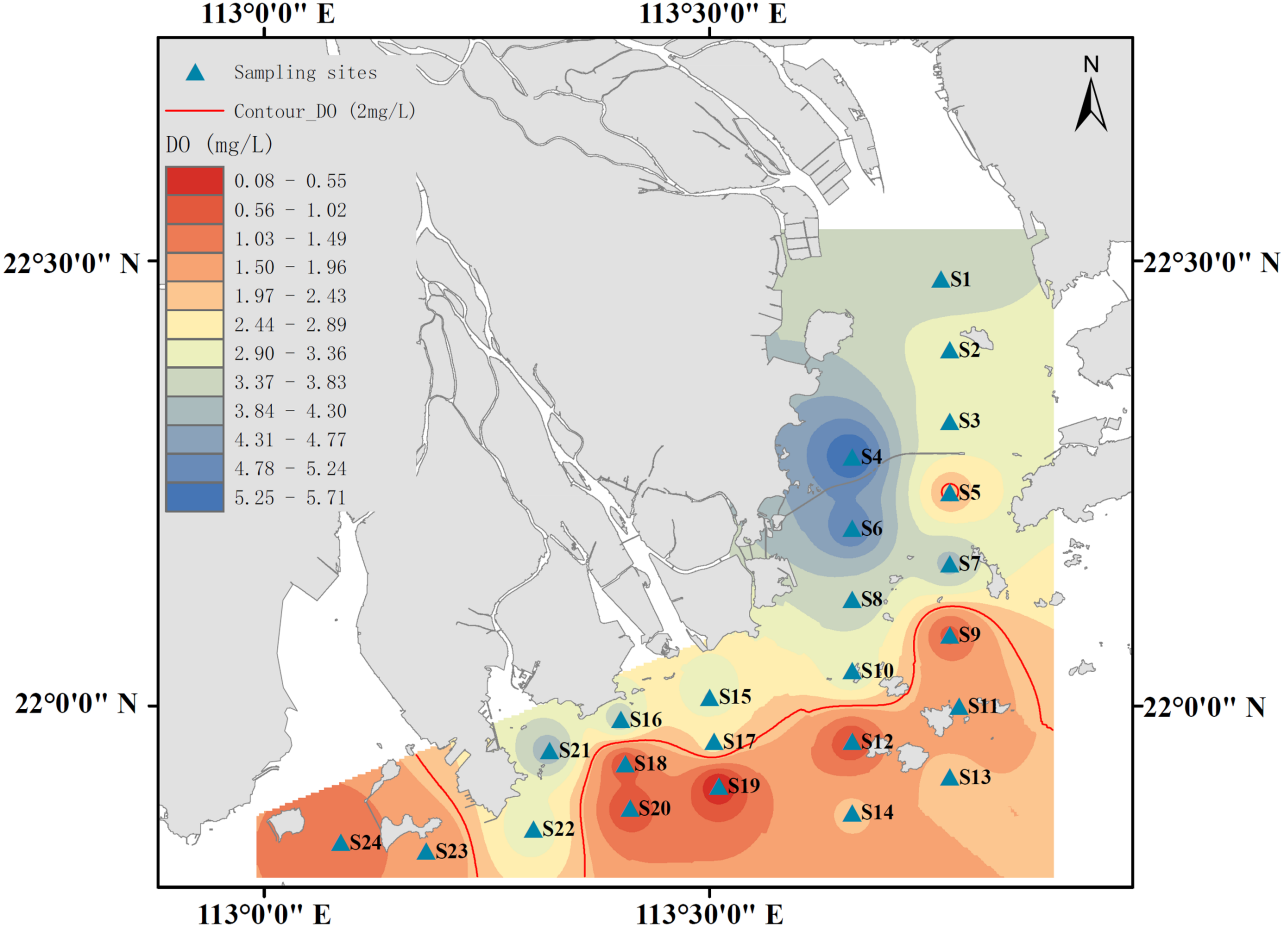
Spatial distribution of bottom dissolved oxygen in the Pearl River Estuary in summer 2021 (The red isodensity line represents the dividing line of dissolved oxygen at 2 mg/L).

In this study, the bottom‐layer dissolved oxygen concentration in the PRE was generally low, with an average concentration of 2.58 mg/L and a maximum value of only 5.71 mg/L (observed at site S4 in shallower water). Large areas of hypoxia (DO ≤ 2 mg/L) were observed within the survey area (Figure 3). Among them, the dissolved oxygen concentrations at sites S5, S9, S11, S12, S13, S14, S18, S19, S20, S23, and S24 were all less than or equal to 2 mg/L. The lowest concentration of dissolved oxygen was recorded at site S19, at only 0.08 mg/L.

### 3.2 Effects of hypoxia on fish community composition and distribution

A total of 104 fish species (2 classes, 12 orders, 46 families, and 82 genera) were collected in this study, indicating high species richness in the PRE. In the hypoxia area (DO ≤ 2 mg/L), 79 fish species (1 class, 9 orders, 39 families, and 64 genera) were collected, accounting for 75.96% of the total species. Among them, 30 species (28.6%) were found exclusively in the hypoxia area. At the order level, the relative abundance of Perciformes and Tetraodontiformes was significantly higher in the hypoxia area than in other areas, while the relative abundance of Clupeiformes and Siluriformes was significantly lower in the hypoxia area. At the family level, the relative abundance of Apogonidae, Tetraodontidae, Siganidae, and Carangidae was significantly higher in the hypoxia area than in other areas, while the relative abundance of Engraulidae, Ariidae, and Clupeidae was significantly lower in the hypoxia area. In terms of species distribution, species such as *Decapterus maruadsi*, *Siganus fuscescens*, *Lagocephalus spadiceus*, and *Ostorhinchus fasciatus* exhibit relatively high relative abundance in the hypoxia area, whereas *Sardinella zunasi*, *Coilia mystus*, *Nuchequula nuchalis*, *Collichthys lucidus*, *Stolephorus commersonnii*, and *Dendrophysa russelii* showed lower relative abundances.

**FIGURE 4:**
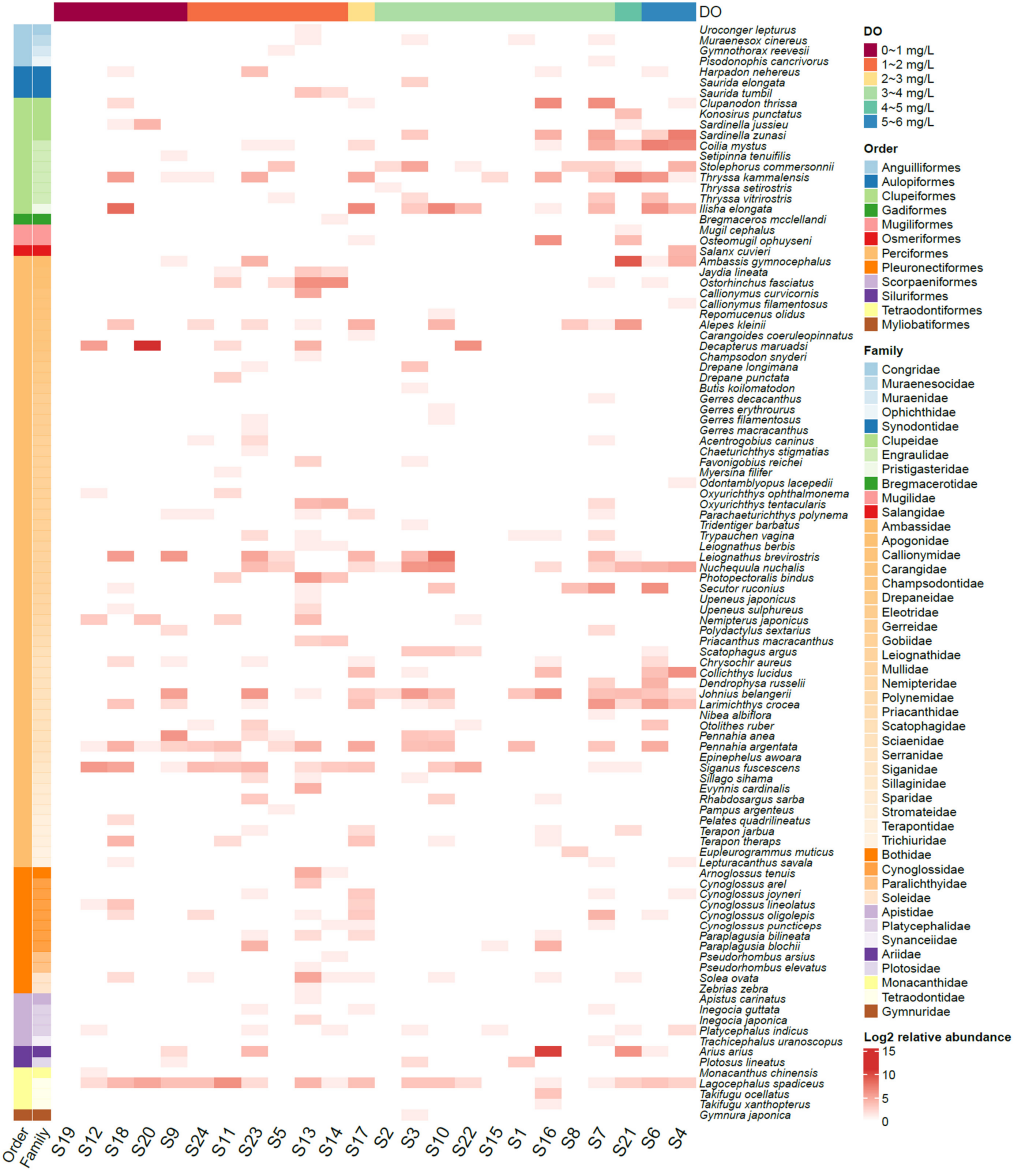
Heatmap illustrating the relative abundance of fish species at different sites in the Pearl River Estuary (Sites are arranged from left to right based on ascending dissolved oxygen concentration. The row annotations on the left use different colors to indicate the order and family of each species. The abundance values in the heatmap have been log‐transformed).

The relative abundance heatmap of fish species at different sites in the PRE (Figure 4) shows that some hypoxic sites with relatively low dissolved oxygen concentrations, such as sites S13, S18, and S23, exhibit high species richness and relative abundance. However, at site S19, which was close to anoxic conditions, the species richness was reduced to zero.

**FIGURE 5:**
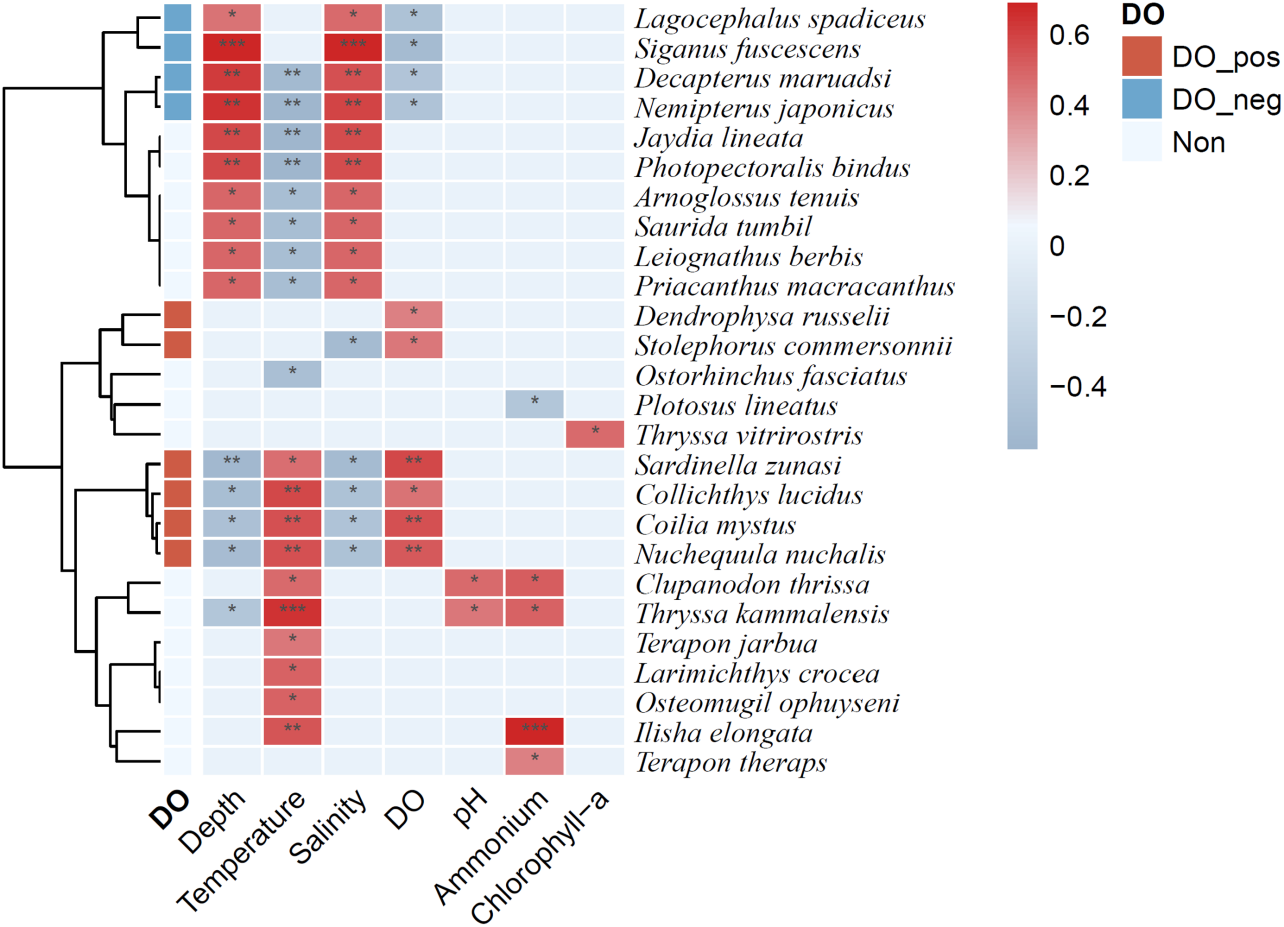
Correlation heatmap between species and environmental factors (Only showing species significantly correlated with environmental factors. “DO_pos,” “DO_neg,” and “Non” indicate species that are positively correlated, negatively correlated, and not correlated with dissolved oxygen, respectively).

The correlation heatmap showed the species significantly (*p* < .05) correlated with environmental factors (Figure 5). It can be observed that depth, temperature, salinity, and dissolved oxygen had relatively large effects on species abundance. Among them, species significantly positively correlated with dissolved oxygen mainly included *Dendrophysa russelii* (*r* = .4205, *p* = .0457), *Stolephorus commersonnii* (*r* = .4455, *p* = .0331), *Sardinella zunasi* (*r* = .5792, *p* = .0038), *Collichthys lucidus* (*r* = .4548, *p* = .0292), *Coilia mystus* (*r* = .5564, *p* = .0058), and *Nuchequula nuchalis* (*r* = .5279, *p* = .0096), suggesting that these species are more sensitive to hypoxia. While species significantly negatively correlated with dissolved oxygen mainly included *Lagocephalus spadiceus* (*r* = −.4580, *p* = .0280), *Siganus fuscescens* (*r* = −.5198, *p* = .0110), *Decapterus maruadsi* (*r* = −.4327, *p* = .0392), and *Nemipterus japonicus* (*r* = −.4444, *p* = .0336), these species were relatively more abundant in the hypoxia area, suggesting that these species are more tolerant to hypoxia.

### 3.3 Effects of hypoxia on fish community diversity

**FIGURE 6:**
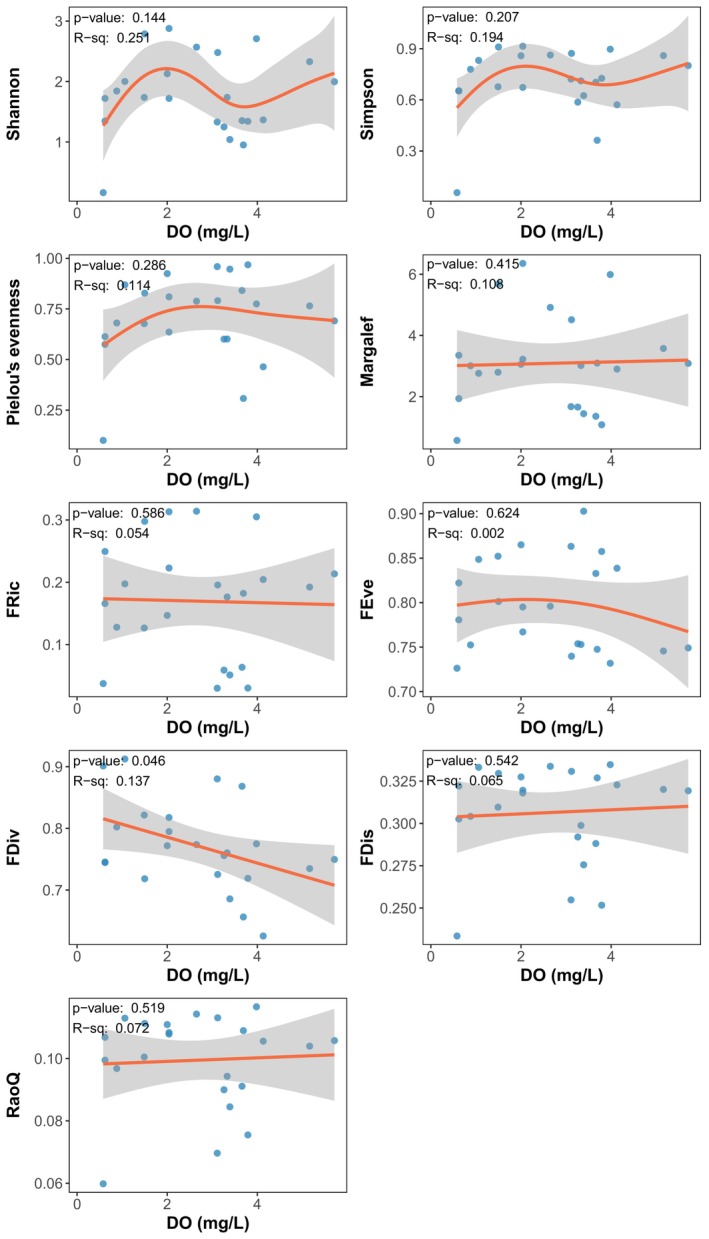
Generalized additive model (GAM) analysis of the relationship between species diversity and functional diversity with dissolved oxygen.

To assess the impact of hypoxia on fish community diversity, species diversity and functional diversity indices were calculated, as shown in Table S3. Generalized additive models (GAMs) were used to perform regression analyses between species diversity indices and environmental factors. The results (Figure 6) indicate that in hypoxia areas (DO ≤ 2 mg/L), there is a non‐significant upward trend (*p* > .05) in fish species diversity indices (Shannon, Simpson, and Pielou evenness indices) with increasing dissolved oxygen levels. Similarly, GAMs were applied to fit regression models for functional diversity indices and environmental factors. The results show that the FDiv index significantly decreases with increasing dissolved oxygen levels (*p* < .05), whereas other functional diversity indices do not show significant changes.

**FIGURE 7:**
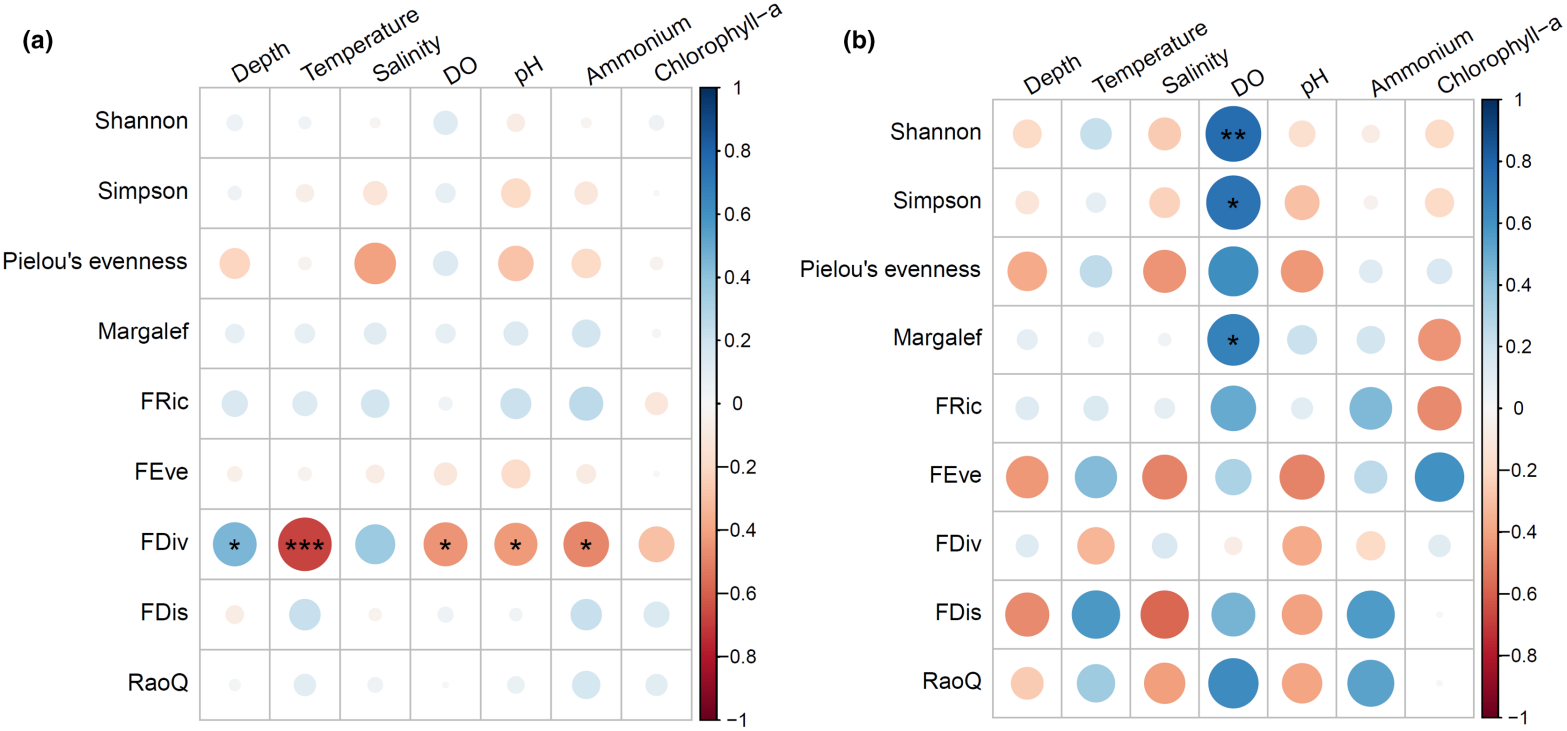
Heatmap of the correlation between fish community diversity and environmental factors. (a) represents the community of all sampling sites (*n* = 24), while (b) represents the community of sites in the hypoxia area (DO ≤ 2 mg/L, *n* = 11).

In order to further verify the impact of hypoxia and other environmental factors on fish community diversity (species diversity and functional diversity), we conducted a correlation analysis between fish community diversity and environmental factors at all sites in the PRE, as well as a separate analysis between fish community diversity and environmental factors at hypoxic sites. The results are shown in Figure 7.

At all sampling sites, there was no significant correlation between species diversity and environmental factors, while in the functional diversity index, the FDiv index was significantly negatively correlated with dissolved oxygen (*r* = −.4483, *p* = .0319). In addition, the FDiv index was significantly negatively correlated with temperature (*r* = −.6754, *p* = .0004), ammonium (*r* = −.4832, *p* = .0195), pH (*r* = −.4294, *p* = .0409), and significantly positively correlated with water depth (*r* = .4536, *p* = .0297).

In the hypoxia area, apart from the FDiv index, both fish species diversity indices and functional diversity indices showed a positive correlation with dissolved oxygen. Specifically, significant positive correlations were found between Shannon (*r* = .7689, *p* = .0057), Simpson (*r* = .7317, *p* = .0162), and Margalef indices (*r* = .6707, *p* = .0338) of species diversity and dissolved oxygen, indicating that dissolved oxygen is the primary limiting factor for fish community diversity in the hypoxia area. These findings suggest that maintaining sufficient dissolved oxygen levels is essential for sustaining fish communities and ecosystem health in the PRE. Higher dissolved oxygen levels provide more favorable environmental conditions for a greater number of species to coexist and exploit different ecological niches, resulting in increased species richness and diversity.

### 3.4 Effects of hypoxia on fish community structure

**FIGURE 8:**
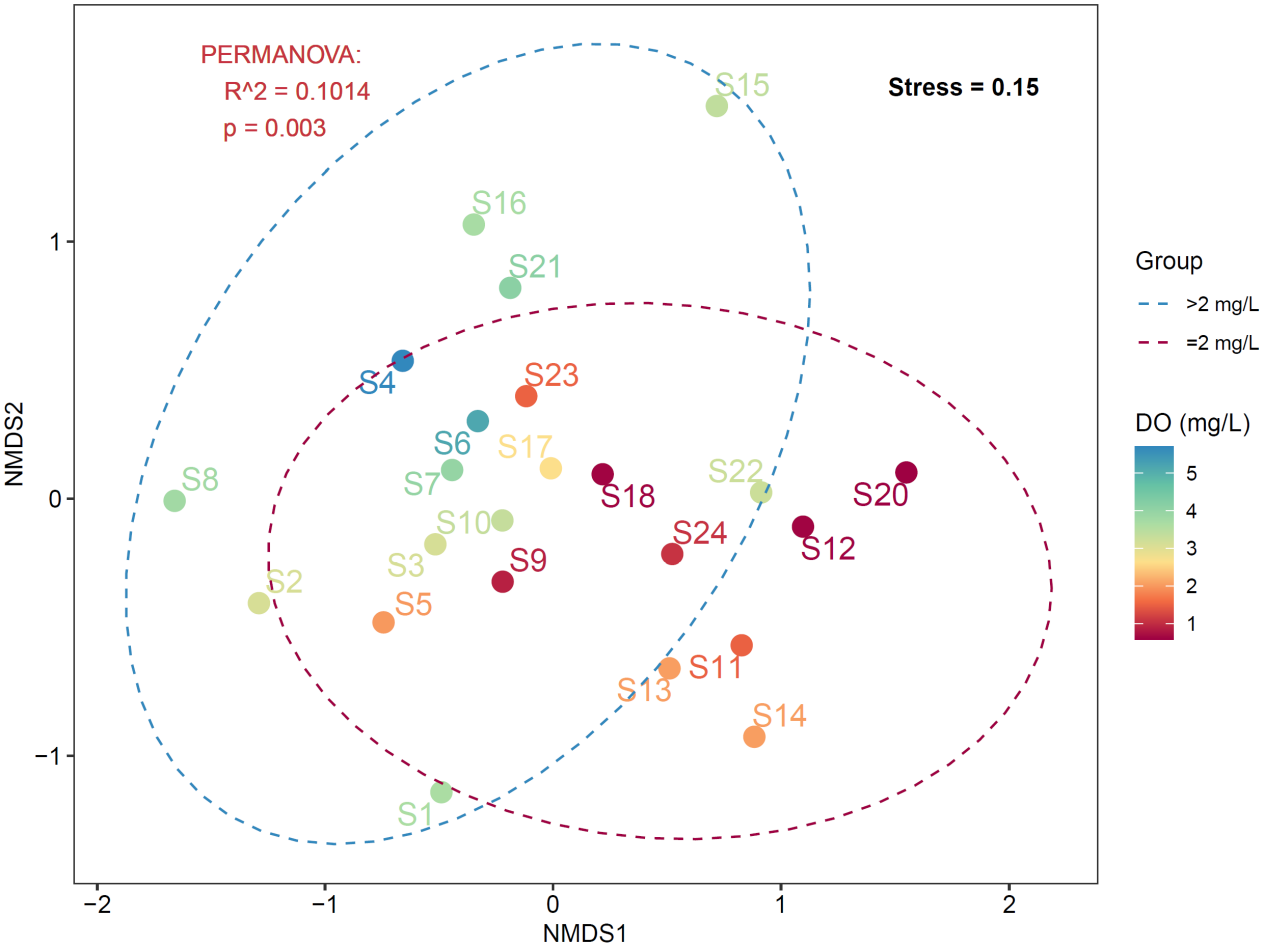
NMDS analysis of fish community structure in the Pearl River Estuary.

Non‐metric Multidimensional Scaling (NMDS) analysis was used to explore differences in fish community structure between hypoxia and normoxia areas (Figure 8). The results indicate that fish communities in hypoxia and normoxia areas can be distinctly classified into two groups. permutational multivariate analysis of variance (PERMANOVA) was used to analyze the explanatory power and significance of community structure differences, showing that there are significant differences in fish community structure between hypoxia and normoxia areas (*R* ^2^ = .1014, *p* = .003).

**FIGURE 9:**
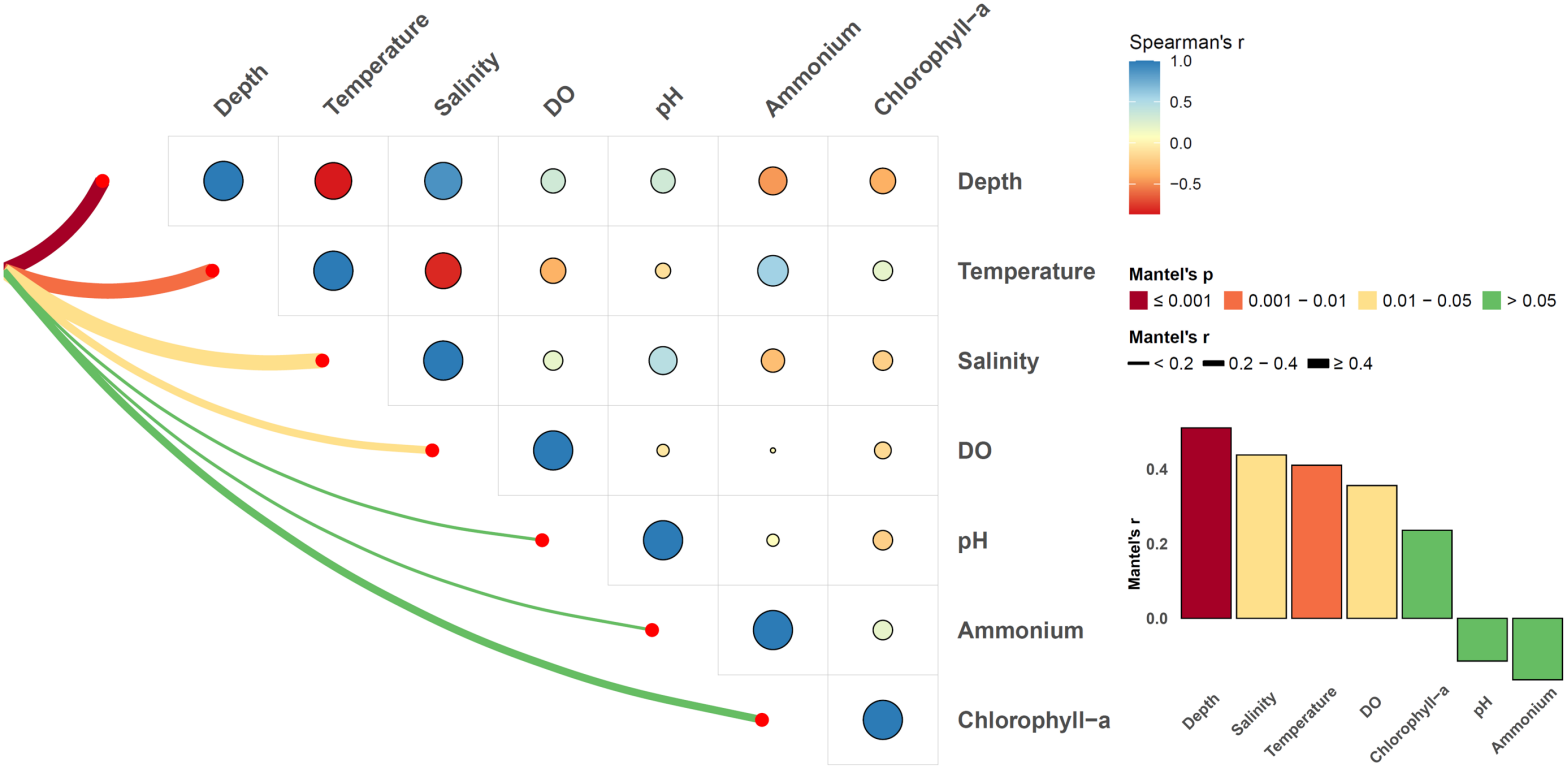
Environmental drivers of demersal fish community structure in the hypoxia area analyzed by the Mantel test based on Spearman's correlation analysis.

To further examine the impact of hypoxia on fish community structure in the PRE, we conducted Mantel tests based on Spearman's correlation analysis to analyze the relationship between fish community structure in hypoxic sites and environmental variables (Figure 9). The results revealed a significant effect of dissolved oxygen on fish community structure in the hypoxia area (*r* = .3560, *p* = .011). Additionally, depth (*r* = .5104, *p* = .001), temperature (*r* = .4105, *p* = .005), and salinity (*r* = .4378, *p* = .011) also exhibited significant impacts on the composition of fish community structure, which may suggest that their synergistic interactions with hypoxia in shaping the overall community assemblage.

## 4 DISCUSSION

In estuarine and marine ecosystems, hypoxia or the decrease in dissolved oxygen levels is one of the environmental conditions that most significantly affects community structure (Breitburg et al., 2018). Hypoxia can reduce the availability and quality of habitat for fish, forcing them to move to more favorable areas or adapt to low‐oxygen conditions (Arend et al., 2011). As a typical subtropical estuary, seasonal hypoxia events occur repeatedly in the land‐sea transition zone of the PRE due to the large amount of nutrient discharged by high‐intensity human activities (Li et al., 2020). In this study, we observed large areas of hypoxia with dissolved oxygen levels below 2 mg/L in the bottom layer of the PRE in summer 2021. The concentration of dissolved oxygen at the bottom was generally low, ranging from 0.08 to 5.71 mg/L. In this regard, we conducted a study on the effects of summer hypoxia on the structure of the demersal fish community in the PRE. The results showed that hypoxia had different degrees of effects on the composition, distribution, diversity, and structure of the PRE fish community. The research results provide important information on the response of demersal fish communities in the PRE to hypoxia. This contribute to our understanding of the impacts of hypoxia on estuarine ecosystems and fisheries resources, and provide a basis for effective management and conservation.

### 4.1 Effects of hypoxia on fish community composition and distribution

Our findings indicated that some moderately hypoxic sites (such as S13, S18, and S23) still maintain high species richness and relative abundance, contrary to the findings of previous studies in other coastal hypoxia areas. For instance, investigation studies on hypoxia areas in the northwestern Gulf of Mexico continental shelf and adjacent waters have shown that fish species richness and total abundance were lowest in hypoxic waters (DO ≤ 1 mg/L), while they increased at moderate dissolved oxygen levels (2–4 mg/L) (Craig & Bosman, 2012).

We propose possible explanations for this phenomenon. The high fish species richness in the hypoxia area of the PRE may be related to its high spatial heterogeneity and primary productivity, which provide shelter and food resources for fish with different adaptation abilities. Influenced by the input of terrestrial substances into the sea, the PRE exhibits extremely high primary productivity, making it an important spawning and nursery ground for various fish species (Liu et al., 2017), and high primary productivity can enhance fish diversity and resources by increasing food availability and trophic complexity. In terms of spatial distribution, sites with high fish species richness in the hypoxia area were mainly concentrated in a few sites adjacent to islands, including S13 and S23 sites, where 35 and 29 fish species were collected, respectively, and the species richness was significantly higher than other sites, while most sites in the hypoxia area had relatively low species richness. Islands exhibit characteristics of high spatial heterogeneity and primary productivity, providing habitats for numerous reef‐associated and reef‐dependent fish species, serving as important locations for foraging and spawning activities (Wu et al., 2022). The islands in the hypoxia area may create spatial refuges and habitats for fish, especially reef‐associated fish that can tolerate low‐oxygen levels. This suggests that islands may play an important role in supporting fishery resources and mitigating the effects of hypoxia on fish by providing alternative resources and niches.

In addition, the spatial distribution and other environmental variables of the sites in the hypoxia area may also have some influence on the fish community composition in the hypoxia area. A study on the fish community structure of the Yangtze River Estuary and adjacent waters also showed that the environmental gradient (water depth, salinity, nutrients, and turbidity) from nearshore to offshore resulted in higher species richness but a lower abundance of fish in the offshore area (Shan et al., 2010). In this survey, the sites in the hypoxia area were mainly distributed in the outer estuarine waters, which may lead to higher species richness.

However, once dissolved oxygen concentrations fall below a certain threshold (exceeding the tolerance limits of fish species), the hypoxia area can significantly impact the fish species composition. For instance, at site S19, where dissolved oxygen was as low as 0.08 mg/L (approaching anoxic conditions), species richness was reduced to 0 and hardly any fish catches were captured during trawl surveys. This result highlights the importance of maintaining adequate oxygen levels to support the richness of fish species within the estuarine ecosystem.

The effects of hypoxia on fish composition are species‐specific, there is a significant variation in tolerance to hypoxic conditions among different species (Bickler & Buck, 2007; Costantini et al., 2008; Gallo & Levin, 2016; Vaquer‐Sunyer & Duarte, 2008). Consequently, some fish species may benefit from hypoxia, as hypoxia‐tolerant species can survive in hypoxic waters, using them as a refuge for feeding or avoiding predators (Altieri, 2008). For example, studies on fish communities along the southeastern coast of China found that the population of hypoxia‐tolerant fish (*Harpadon nehereus*) increased dramatically and dominated the community due to the decrease in dissolved oxygen (Kang et al., 2021). In Chesapeake Bay, eutrophication‐driven bottom hypoxia resulted in zooplankton tending to use hypoxia areas as a refuge, benefiting demersal fish with higher hypoxia tolerance due to the increase in their food source from zooplankton (Ludsin et al., 2009). Research on deep sea fish communities in the oxygen minimum zone of the Gulf of California has shown that, although species diversity decreases in low‐oxygen environments, some species exhibit adaptations that enable them to survive under hypoxic conditions (Gallo et al., 2020). Conversely, some fish species are negatively affected by hypoxia. In the Gulf of Mexico, there is a significant difference in fish composition between hypoxic and non‐hypoxic zones, including many fish species such as the recreational or commercial fish species *Micropogonias undulatus* and *Lutjanus campechanus*, with significantly lower catch per unit effort (CPUE) in hypoxic zones compared to non‐hypoxic zones (Glaspie et al., 2019). Additionally, hypoxic events can induce population shifts in fish species through extreme mortality effects, as exemplified by the mass death of flatfish species when low‐oxygen water masses upwell onto the continental shelf in Oregon's marine waters (Grantham et al., 2004).

In this study, we observed varied responses among fish species to hypoxia, evident from their correlations with dissolved oxygen levels. Some species, such as *Dendrophysa russelii*, *Stolephorus commersonnii*, *Sardinella zunasi*, *Collichthys lucidus*, *Coilia mystus*, and *Nuchequula nuchalis*, have a significantly positive correlation with dissolved oxygen, suggesting that these species are more sensitive to hypoxia and prefer higher oxygen levels. Due to their strong migration ability, fish can make a sensitive response to environmental changes by avoiding unfavorable conditions. Some studies have shown that when there is hypoxia in the bottom waters, fish can move upward to escape the hypoxia area (Eby & Crowder, 2002). Studies on the bay anchovy (*Anchoa mitchilli*), a species of the Clupeiformes order in Chesapeake Bay, also indicate that it was forced to vertically migrate into oxygen‐rich surface waters during the summer due to the influence of hypoxia, but this behavior increases the risk of predation (Costantini et al., 2008). In this study, *Stolephorus commersonnii*, *Sardinella zunasi*, and *Coilia mystus* are all species of the order Clupeiformes, hypoxia area in the bottom water may force these species to migrate to surface waters with higher dissolved oxygen, leading to habitat compression. Other species, such as *Lagocephalus spadiceus*, *Siganus fuscescens*, *Decapterus maruadsi*, and *Nemipterus japonicus*, increased in relative abundance under low‐oxygen conditions, indicating that they are more tolerant to hypoxia and can survive at lower oxygen levels, and these species may utilize the bottom layer of hypoxia area as a feeding or refuge area. In addition, in terms of other abiotic and biotic factors (such as temperature, salinity, and predation risk), entering a suboptimal new area may come at a high cost, so staying in hypoxic waters may be more advantageous (Eby et al., 2005; Eby & Crowder, 2002; Kramer, 1987). This may also be one of the reasons for the higher abundance of certain species in hypoxia area.

In summary, hypoxia can cause shifts in fish community composition by selecting for more tolerant species and excluding more sensitive species. Although the decrease in bottom dissolved oxygen does act as a filter for species composition, once it exceeds a certain threshold, all fish species will be adversely affected (Shinohara et al., 2022). Additionally, it is undeniable that our current investigation, limited by its timeframe, only provides a transient snapshot of the community composition. Fish exhibit strong migratory abilities, resulting in dynamic distribution patterns, and the fluctuating nature of dissolved oxygen concentration may also be a significant factor that cannot be ignored. This dynamism may explain the higher‐than‐expected species abundance or diversity observed at some hypoxic sites. Therefore, to gain a more comprehensive understanding of the effects of hypoxia on fish community composition and distribution, future research should involve longer‐term continuous observations of both fish communities and the hypoxic environment.

### 4.2 Effects of hypoxia on fish community structure and diversity

The analysis of fish community diversity indices reveals a complex relationship between hypoxia and fish diversity. Generalized additive models (GAM) and correlation analyses indicate that dissolved oxygen levels play a crucial role in shaping both species and functional diversity in the PRE. The correlation analysis between community diversity and environmental factors revealed that although there was no significant correlation between species diversity and environmental factors when considering all sampling sites, a significant positive correlation was observed between fish species diversity indices (Shannon, Simpson, and Margalef) and dissolved oxygen when only considering the sites in the hypoxia area.

On the one hand, the above results highlight that dissolved oxygen plays a critical role in shaping the diversity of fish communities. However, on the other hand, the results also reflect that the relationship between fish community diversity and dissolved oxygen levels was nonlinear, indicating that the effect of dissolved oxygen on the fish community structure at low‐oxygen sites was markedly stronger than that on the overall fish community structure in the PRE.

We propose several possible explanations for this nonlinear relationship. First, the increase in species diversity with higher dissolved oxygen levels can be explained by the fact that higher dissolved oxygen levels provide more favorable environmental conditions for a greater number of species to coexist and exploit different ecological niches, resulting in increased species richness and diversity. However, when dissolved oxygen levels exceed a certain threshold (around 2 mg/L), the effect of dissolved oxygen on species diversity may become weaker or negligible, as other environmental factors (such as depth, temperature, and salinity) may become more important in determining species distribution and abundance. Several other studies have reported similar findings, noting that the species diversity of demersal fish typically decreases with lower dissolved oxygen levels, but this effect is threshold‐dependent. For example, in the hypoxia areas of Oregon and the entire US Pacific coast, species richness is significantly positively correlated with near‐bottom dissolved oxygen concentrations, while at lower oxygen concentrations, the near‐bottom dissolved oxygen concentration has a greater impact on species richness (Keller et al., 2010, 2015). Another study of deep sea fish communities in the Gulf of California also demonstrated a significant threshold response of diversity to oxygen levels, with diversity sharply declining once oxygen levels dropped below a certain point (Gallo et al., 2020). These studies all suggest that the response of fish communities to dissolved oxygen concentrations is nonlinear.

However, it is worth noting that the FDiv index generally decreased with higher dissolved oxygen levels, indicating a potential shift in the functional traits and ecological roles of fish species in response to changing oxygen conditions. The size of the FDiv index reflects the degree of functional divergence of species in the community, the higher the FDiv index, the greater the difference in functional traits of species within the community, the stronger the ecological niche complementarity, and the weaker the competition (Villeger et al., 2008). Therefore, the decrease in dissolved oxygen may, to some extent, reduce ecological niche complementarity, and increase competition among fish communities. In addition, the FDiv index was also significantly correlated with environmental variables such as water depth and temperature, suggesting that the spatial gradients and interactions of these environmental factors may be one of the important reasons for the decline of the FDiv index. In this study, the sites in the hypoxia area were mainly distributed in the outer estuarine waters, where the bottom‐layer environment had relatively low variability, which may lead to a higher FDiv index. Some other studies also suggested that the high variability of the inner estuarine environment resulted in only a few dominant species that could adapt, thus lowering the FDiv value (Zhou et al., 2019). A study on the fish community of the Paraiba estuary in Brazil showed that the FDiv index increased downstream and remained at a high level in the area near the open sea (Dolbeth et al., 2016).

Mantel tests provide additional insights into the relationship between fish community structure and environmental factors, including dissolved oxygen. The significant effect of dissolved oxygen on fish community structure in the hypoxia area, as indicated by the Mantel test, highlights its importance in shaping the structure of fish communities. Additionally, depth, temperature, and salinity also significantly affected the composition of the fish community. These factors may affect fish community structure by influencing the habitat suitability, physiological tolerance, and food availability of different fish species.

These results indicate that, in addition to dissolved oxygen, other environmental factors also play an important role in shaping the fish community structure of the hypoxia area in the PRE. Hypoxia alone may lead to shifts in species composition and abundance, but when interacting with other environmental variables, the magnitude and direction of these changes may be amplified or mitigated (Gobler & Baumann, 2016). For example, temperature has an important effect on the formation of hypoxia and the structure of fish communities in hypoxic areas. On the one hand, elevated temperatures can lead to increased oxygen consumption and reduced oxygen solubility, exacerbating hypoxia. The existence of thermocline in water inhibits the vertical mixing and exchange of dissolved oxygen in the water column, resulting in oxygen depletion in the bottom layer, which is one of the important reasons for the formation of hypoxia (Keller et al., 2015; Virtanen et al., 2019). On the other hand, higher temperatures increase organisms' metabolic demand for oxygen, thereby increasing their vulnerability to hypoxia (Pörtner & Knust, 2007; Vaquer‐Sunyer & Duarte, 2011). Therefore, temperature is also one of the important factors affecting the structure of fish communities in the hypoxia area. These findings highlight that understanding the complex interplay of multiple environmental factors is crucial for a comprehensive assessment of the effects of hypoxia on fish communities.

## 5 CONCLUSIONS

This study investigated the effects of hypoxia on the demersal fish community structure in the PRE during summer 2021, a typical hypoxic period in this region. Hypoxia had significant effects on the composition, diversity, and structure of the fish community in the PRE, and different fish species showed different tolerance and adaptation strategies to cope with hypoxia. Maintaining adequate levels of dissolved oxygen is essential for sustaining fish diversity and maintaining a healthy fish community in hypoxic environments. The interaction of dissolved oxygen and other environmental factors such as water depth, temperature, and salinity influence the fish community structure in the hypoxia area. Our findings provide new insights into the responses of fish communities to hypoxia in estuarine ecosystems and have implications for the ecological health and management of the PRE. However, the formation and maintenance of hypoxic conditions in the PRE are dynamic processes. In addition, under natural conditions, hypoxia is usually associated with a variety of complex factors. The inability to separate the effects of hypoxia from the interactions of these complex factors makes it difficult to attribute many of the observed ecological effects to hypoxia (Mandic & Regan, 2018; Wu, 2002). Therefore, research on the effects of hypoxia on the structure of fish communities still faces certain limitations and challenges. Further research is needed to delve deeper into the underlying mechanisms driving these effects and assess the potential long‐term impacts of hypoxia on the estuarine fish community.

## AUTHOR CONTRIBUTIONS

**Han Lai:** Conceptualization (lead); data curation (lead); formal analysis (lead); investigation (lead); methodology (lead); software (lead); visualization (lead); writing – original draft (lead); writing – review and editing (lead). **Sheng Bi:** Investigation (supporting); methodology (supporting); validation (supporting). **Huadong Yi:** Investigation (supporting); methodology (supporting); validation (supporting). **Haiyang Li:** Investigation (supporting); validation (supporting). **Xuchong Wei:** Investigation (supporting); validation (supporting). **Gongpei Wang:** Investigation (supporting); validation (supporting). **Dingli Guo:** Investigation (supporting); validation (supporting). **Xuange Liu:** Validation (supporting). **Jiahui Chen:** Validation (supporting). **Qiuxian Chen:** Validation (supporting). **Zhilun Zhang:** Validation (supporting). **Shuang Liu:** Validation (supporting). **Chenlei Huang:** Validation (supporting). **Li Lin:** Validation (supporting). **Guifeng Li:** Conceptualization (equal); funding acquisition (lead); project administration (lead); resources (equal); supervision (equal); validation (equal); writing – review and editing (equal).

## CONFLICT OF INTEREST STATEMENT

The authors declare that they have no known competing financial interests or personal relationships that could have appeared to influence the work reported in this paper.

### OPEN RESEARCH BADGES

This article has earned an Open Data badge for making publicly available the digitally‐shareable data necessary to reproduce the reported results. The data is available at [https://doi.org/10.5281/zenodo.12156555].

## Supporting information


Data S1

## Data Availability

The data that supports the findings of this study are available in the supplementary material of this article. All original code is available at: https://doi.org/10.5281/zenodo.12156555.
