# Supplementary material for: Characteristics of demersal fish community structure during summer hypoxia in the Pearl River Estuary, China
Source: Ecol Evol. 2024 Jul 11;14(7):e11722. doi: 10.1002/ece3.11722 (PMC11237343; doi:10.1002/ece3.11722)
Supplement: Supplementary file 1 — Data S1 [file ECE3-14-e11722-s001.docx]

**Title: Characteristics of demersal fish community structure during summer hypoxia in the Pearl River Estuary, China**


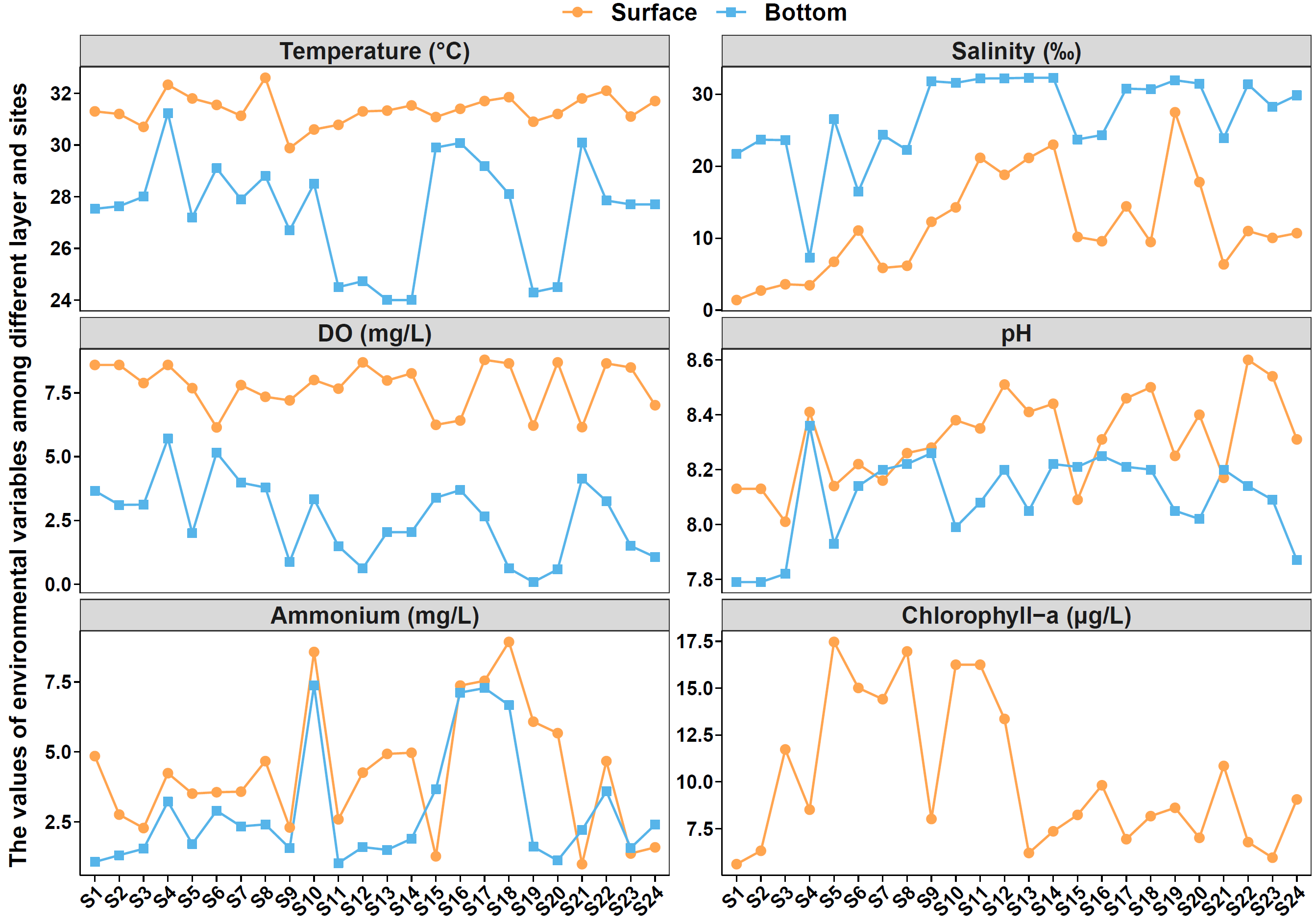


Figure S1. Distribution of environmental factors at different sampling sites.


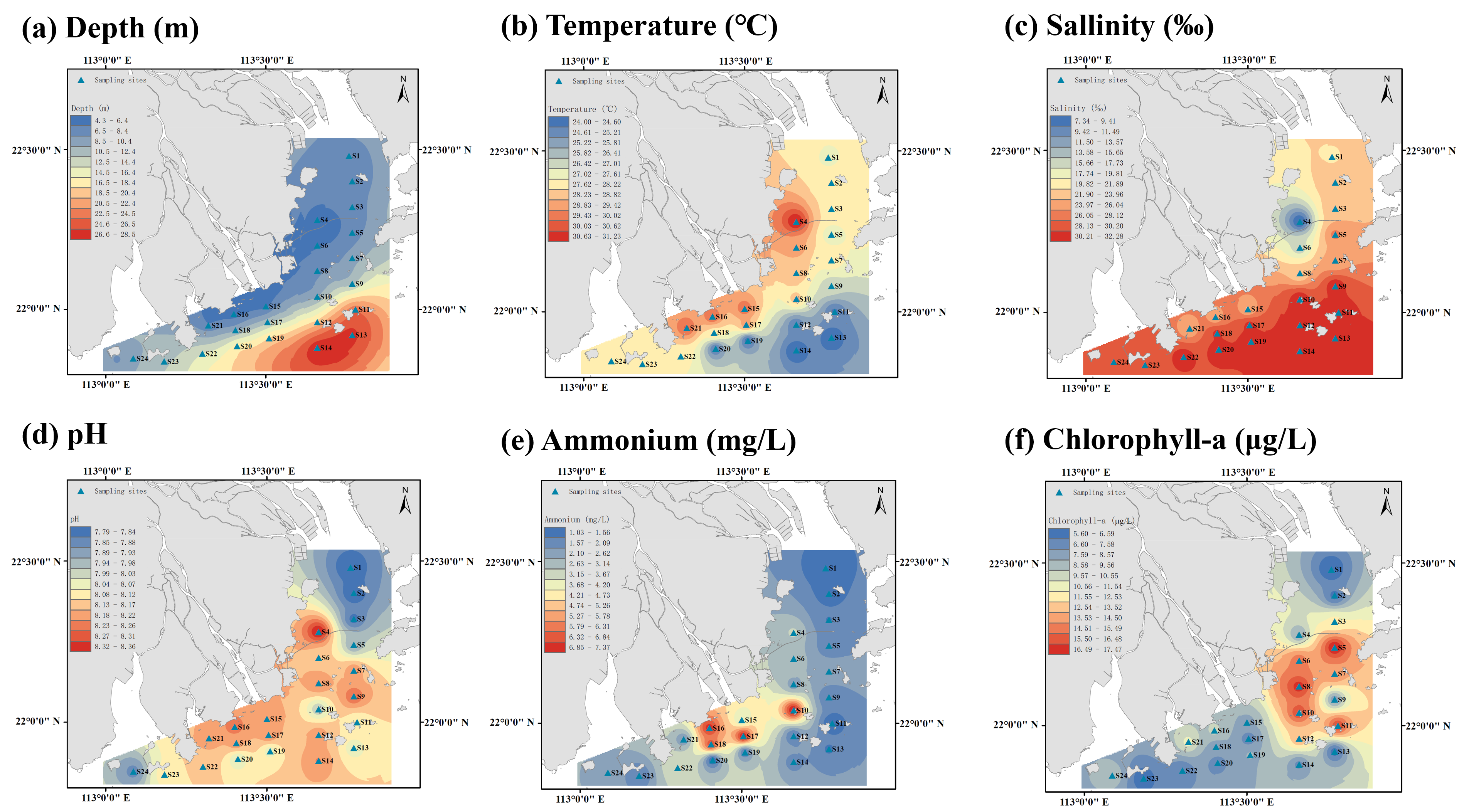


Figure S2. Spatial distribution of environmental variables in the Pearl River Estuary.

Table S1. Formulas used to calculate the species diversity index.

| Diversity indices | Formula | Description |
| --- | --- | --- |
| Shannon-Wiener Diversity Index (H) | 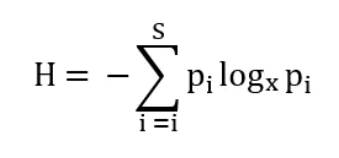 | where **S** is the species richness index of the community, **P_i_**_​_​ is the relative abundance of species **i**, and **x** is typically the base of 2 or *e*. |
| Gini-Simpson Index (GS) | 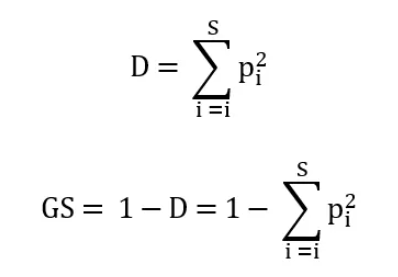 | where **S** is the species richness index of the community, and **P_i_**_​_ is the relative abundance of species **i**. |
| Margalef Richness Index (D) | 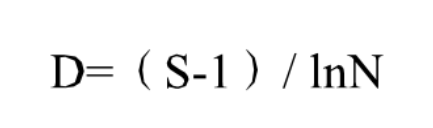 | where **S** is the species richness index of the community, and **N** is the total number of observed individuals. |
| Pielou Evenness Index (J) | 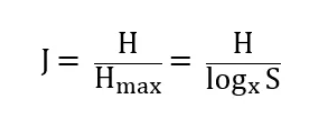 | where **H** is the Shannon index, **H_max_**​ is the maximum Shannon index that can be achieved with the same species richness, **S** is the species richness index of the community, and **x** is typically the base of 2 or *e*. |

Table S2. Functional traits of fishes in the Pearl River Estuary.

| **Species** | **Body form** | **Position of mouth** | **Swimming mode** | **Length at maturity (cm)** | **Age at maturity (year)** | **Life span** | **EUFG** | **Trophic guild** | **Trophic level** | **Mean**  **temperature preference (℃)** |
| --- | --- | --- | --- | --- | --- | --- | --- | --- | --- | --- |
| *Acentrogobius caninus* | anguilliform | terminal | diodontiform | 11.9 | 1.9 | Medium | DI | Zoobenthivore | 3.50 | 28.2 |
| *Alepes kleinii* | compressiform | terminal | carangiform | 10.7 | 0.9 | Short | MS | Planktivore | 3.54 | 27.9 |
| *Ambassis gymnocephalus* | fusiform | terminal | carangiform | 12.0 | 0.6 | Short | DI | Miscellaneous | 3.91 | 28.1 |
| *Apistus carinatus* | fusiform | terminal | subcarangiform | 12.9 | 1.5 | Medium | MS | Zoobenthivore | 3.61 | 28.0 |
| *Arius arius* | anguilliform | sub-terminal/inferior | carangiform | 23.8 | 3.0 | Long | DI | Zoobenthivore | 3.50 | 28.5 |
| *Arnoglossus tenuis* | compressiform_flat | terminal | anguilliform | 8.2 | 2.1 | Medium | MS | Zoobenthivore | 3.50 | 23.3 |
| *Bregmaceros mcclellandi* | anguilliform | terminal | subcarangiform | 6.7 | 1.5 | Medium | MM | Planktivore | 3.30 | 8.8 |
| *Butis koilomatodon* | fusiform | terminal | subcarangiform | 7.4 | 1.0 | Short | DI | Zoobenthivore | 4.01 | 28.3 |
| *Callionymus curvicornis* | depressiform | sub-terminal/inferior | subcarangiform | 7.7 | 1.4 | Short | MS | Zoobenthivore | 3.24 | 21.8 |
| *Callionymus filamentosus* | depressiform | sub-terminal/inferior | subcarangiform | 12.9 | 1.8 | Medium | MS | Zoobenthivore | 3.27 | 24.0 |
| *Carangoides coeruleopinnatus* | compressiform | terminal | carangiform | 24.4 | 1.6 | Medium | MS | Piscivore | 4.44 | 27.8 |
| *Chaeturichthys stigmatias* | anguilliform | terminal | diodontiform | 17.5 | 2.7 | Long | MS | Zoobenthivore | 3.84 | 26.6 |
| *Champsodon snyderi* | anguilliform | superior | subcarangiform | 7.0 | 1.3 | Short | MS | Piscivore | 4.02 | 20.7 |
| *Chrysochir aureus* | fusiform | sub-terminal/inferior | labriform | 18.5 | 1.6 | Medium | MS | Zoobenthivore | 3.50 | 28.3 |
| *Clupanodon thrissa* | compressiform | terminal | carangiform | 12.5 | 1.6 | Medium | DI | Planktivore | 3.14 | 27.4 |
| *Coilia mystus* | compressiform | sub-terminal/inferior | subcarangiform | 12.9 | 1.3 | Short | DI | Planktivore | 3.20 | 26.4 |
| *Collichthys lucidus* | fusiform | terminal | labriform | 12.8 | 1.2 | Short | MM | Miscellaneous | 3.64 | 27.4 |
| *Cynoglossus arel* | compressiform_flat | sub-terminal/inferior | anguilliform | 31.5 | 2.8 | Long | MS | Zoobenthivore | 3.46 | 27.7 |
| *Cynoglossus joyneri* | compressiform_flat | sub-terminal/inferior | anguilliform | 17.2 | 3.8 | Long | MS | Zoobenthivore | 4.32 | 27.2 |
| *Cynoglossus lineolatus* | compressiform_flat | sub-terminal/inferior | anguilliform | 6.9 | 0.8 | Short | MS | Zoobenthivore | 3.31 | 27.9 |
| *Cynoglossus oligolepis* | compressiform_flat | sub-terminal/inferior | anguilliform | 20.8 | 2.2 | Medium | MS | Zoobenthivore | 3.53 | 28.2 |
| *Cynoglossus puncticeps* | compressiform_flat | sub-terminal/inferior | anguilliform | 14.8 | 1.2 | Short | MM | Zoobenthivore | 3.27 | 28.0 |
| *Decapterus maruadsi* | fusiform | terminal | carangiform | 15.8 | 0.8 | Short | MS | Planktivore | 3.40 | 28.0 |
| *Dendrophysa russelii* | fusiform | sub-terminal/inferior | labriform | 11.5 | 0.8 | Short | DI | Zoobenthivore | 3.58 | 28.3 |
| *Drepane longimana* | compressiform | terminal | labriform | 29.0 | 3.2 | Long | DI | Zoobenthivore | 3.50 | 28.2 |
| *Drepane punctata* | compressiform | terminal | labriform | 26.3 | 3.6 | Long | DI | Zoobenthivore | 3.32 | 28.2 |
| *Epinephelus awoara* | fusiform | terminal | subcarangiform | 34.1 | 3.6 | Long | MS | Piscivore | 3.64 | 22.5 |
| *Eupleurogrammus muticus* | compressiform | superior | amiiform | 47.4 | 1.5 | Medium | MS | Piscivore | 4.08 | 28.4 |
| *Evynnis cardinalis* | compressiform | terminal | subcarangiform | 23.8 | 2.9 | Long | MS | Zoobenthivore | 3.27 | 25.2 |
| *Favonigobius reichei* | anguilliform | terminal | diodontiform | 5.9 | 1.0 | Short | DI | Miscellaneous | 3.46 | 28.5 |
| *Gerres decacanthus* | compressiform | terminal | carangiform | 6.4 | 0.7 | Short | MM | Zoobenthivore | 3.24 | 28.6 |
| *Gerres erythrourus* | compressiform | terminal | carangiform | 18.5 | 1.5 | Medium | MM | Zoobenthivore | 3.34 | 28.5 |
| *Gerres filamentosus* | compressiform | terminal | carangiform | 23.5 | 1.8 | Medium | DI | Zoobenthivore | 3.34 | 28.2 |
| *Gerres macracanthus* | compressiform | terminal | carangiform | 18.5 | 1.5 | Medium | MM | Zoobenthivore | 3.38 | 28.4 |
| *Gymnothorax reevesii* | anguilliform_eel | terminal | anguilliform | 39.1 | 3.4 | Long | MS | Piscivore | 3.99 | 28.0 |
| *Gymnura japonica* | depressiform | sub-terminal/inferior | rajiform | 53.5 | 3.4 | Long | MS | Piscivore | 3.78 | 28.1 |
| *Harpadon nehereus* | anguilliform | terminal | carangiform | 14.8 | 0.5 | Short | MM | Piscivore | 4.08 | 21.2 |
| *Ilisha elongata* | compressiform | superior | carangiform | 31.9 | 1.8 | Medium | MM | Miscellaneous | 3.79 | 27.9 |
| *Inegocia guttata* | depressiform | terminal | subcarangiform | 29.0 | 2.9 | Long | MM | Piscivore | 3.86 | 25.2 |
| *Inegocia japonica* | depressiform | terminal | subcarangiform | 21.2 | 2.2 | Medium | MS | Piscivore | 3.69 | 27.9 |
| *Jaydia lineata* | fusiform | terminal | carangiform | 5.8 | 0.8 | Short | MM | Zoobenthivore | 3.65 | 26.4 |
| *Johnius belangerii* | fusiform | sub-terminal/inferior | labriform | 18.5 | 1.3 | Medium | DI | Zoobenthivore | 3.27 | 28.2 |
| *Konosirus punctatus* | compressiform | sub-terminal/inferior | carangiform | 18.1 | 2.9 | Long | MM | Planktivore | 2.94 | 20.3 |
| *Lagocephalus spadiceus* | anguilliform | terminal | tetraodontiform | 22.5 | 1.7 | Medium | MM | Miscellaneous | 3.66 | 26.8 |
| *Larimichthys crocea* | fusiform | terminal | labriform | 30.6 | 2.3 | Medium | MM | Miscellaneous | 3.72 | 22.8 |
| *Leiognathus berbis* | compressiform | terminal | carangiform | 7.6 | 0.7 | Short | MS | Zoobenthivore | 3.31 | 28.0 |
| *Leiognathus brevirostris* | compressiform | terminal | carangiform | 9.4 | 0.5 | Short | DI | Omnivore | 2.96 | 28.2 |
| *Lepturacanthus savala* | compressiform | superior | amiiform | 56.0 | 0.8 | Short | MS | Piscivore | 4.31 | 28.3 |
| *Monacanthus chinensis* | compressiform | terminal | balistiform | 22.8 | 2.6 | Long | MM | Omnivore | 2.36 | 28.0 |
| *Mugil cephalus* | fusiform | sub-terminal/inferior | subcarangiform | 32.7 | 2.2 | Medium | DI | Omnivore | 2.48 | 23.2 |
| *Muraenesox cinereus* | anguilliform_eel | terminal | anguilliform | 64.3 | 3.2 | Long | MM | Piscivore | 4.38 | 17.4 |
| *Myersina filifer* | anguilliform | terminal | diodontiform | 8.9 | 1.5 | Medium | MS | Omnivore | 3.41 | 28.2 |
| *Nemipterus japonicus* | fusiform | terminal | carangiform | 15.8 | 1.0 | Short | MS | Miscellaneous | 4.12 | 28.4 |
| *Nibea albiflora* | fusiform | sub-terminal/inferior | labriform | 25.6 | 1.1 | Short | MS | Miscellaneous | 4.01 | 23.5 |
| *Nuchequula nuchalis* | compressiform | terminal | carangiform | 15.7 | 1.4 | Medium | MM | Zoobenthivore | 2.97 | 22.4 |
| *Odontamblyopus lacepedii* | anguilliform | terminal | diodontiform | 18.7 | 3.4 | Long | MM | Miscellaneous | 3.89 | 28.4 |
| *Osteomugil ophuyseni* | fusiform | terminal | subcarangiform | 12.1 | 1.8 | Medium | DI | Planktivore | 2.41 | 28.5 |
| *Ostorhinchus fasciatus* | fusiform | terminal | carangiform | 8.6 | 1.1 | Short | MS | Zoobenthivore | 3.63 | 28.1 |
| *Otolithes ruber* | fusiform | terminal | labriform | 26.5 | 1.5 | Medium | DI | Piscivore | 3.60 | 28.3 |
| *Oxyurichthys ophthalmonema* | anguilliform | terminal | diodontiform | 11.8 | 2.0 | Medium | ES | Miscellaneous | 3.84 | 28.4 |
| *Oxyurichthys tentacularis* | anguilliform | terminal | diodontiform | 11.2 | 1.8 | Medium | DI | Miscellaneous | 4.20 | 28.2 |
| *Pampus argenteus* | compressiform | sub-terminal/inferior | carangiform | 19.0 | 1.3 | Medium | MM | Miscellaneous | 3.30 | 28.1 |
| *Parachaeturichthys polynema* | anguilliform | terminal | diodontiform | 10.0 | 1.7 | Medium | MM | Zoobenthivore | 3.06 | 26.5 |
| *Paraplagusia bilineata* | compressiform_flat | sub-terminal/inferior | anguilliform | 19.4 | 1.7 | Medium | MM | Zoobenthivore | 3.47 | 28.5 |
| *Paraplagusia blochii* | compressiform_flat | sub-terminal/inferior | anguilliform | 14.1 | 1.3 | Short | MS | Zoobenthivore | 3.41 | 28.2 |
| *Pelates quadrilineatus* | fusiform | terminal | subcarangiform | 18.5 | 1.8 | Medium | MM | Zoobenthivore | 3.59 | 28.0 |
| *Pennahia anea* | fusiform | terminal | labriform | 20.9 | 1.3 | Medium | MS | Miscellaneous | 3.99 | 28.3 |
| *Pennahia argentata* | fusiform | terminal | labriform | 26.2 | 1.6 | Medium | MM | Miscellaneous | 4.06 | 20.9 |
| *Photopectoralis bindus* | compressiform | terminal | carangiform | 10.0 | 1.4 | Short | DI | Zoobenthivore | 2.93 | 28.0 |
| *Pisodonophis cancrivorus* | anguilliform_eel | terminal | anguilliform | 57.3 | 2.9 | Long | DI | Zoobenthivore | 3.82 | 28.4 |
| *Platycephalus indicus* | depressiform | terminal | subcarangiform | 35.0 | 1.8 | Medium | MM | Piscivore | 3.60 | 26.5 |
| *Plotosus lineatus* | anguilliform | sub-terminal/inferior | anguilliform | 16.9 | 1.7 | Medium | DI | Zoobenthivore | 3.57 | 28.1 |
| *Polydactylus sextarius* | fusiform | sub-terminal/inferior | carangiform | 11.5 | 2.1 | Medium | DI | Miscellaneous | 3.79 | 27.7 |
| *Priacanthus macracanthus* | fusiform | superior | subcarangiform | 15.0 | 0.7 | Short | MM | Miscellaneous | 4.11 | 24.4 |
| *Pseudorhombus arsius* | compressiform_flat | terminal | anguilliform | 25.0 | 4.3 | Long | MM | Zoobenthivore | 4.16 | 27.0 |
| *Pseudorhombus elevatus* | compressiform_flat | terminal | anguilliform | 24.0 | 0.7 | Short | MS | Zoobenthivore | 3.50 | 26.9 |
| *Repomucenus olidus* | depressiform | sub-terminal/inferior | subcarangiform | 7.7 | 3.1 | Medium | ES | Planktivore | 3.34 | 26.5 |
| *Rhabdosargus sarba* | compressiform | terminal | subcarangiform | 44.0 | 2.1 | Medium | MM | Zoobenthivore | 3.25 | 28.0 |
| *Salanx cuvieri* | anguilliform | terminal | subcarangiform | 10.2 | 0.8 | Short | DI | Planktivore | 2.81 | 27.0 |
| *Sardinella jussieu* | compressiform | terminal | carangiform | 11.3 | 2.1 | Medium | MM | Planktivore | 2.87 | 27.3 |
| *Sardinella zunasi* | compressiform | terminal | carangiform | 10.0 | 1.3 | Short | MM | Omnivore | 3.17 | 19.7 |
| *Saurida elongata* | anguilliform | terminal | carangiform | 21.7 | 0.6 | Short | MS | Piscivore | 4.50 | 27.4 |
| *Saurida tumbil* | anguilliform | terminal | carangiform | 34.8 | 2.6 | Long | DI | Piscivore | 4.40 | 27.9 |
| *Scatophagus argus* | compressiform | terminal | labriform | 15.0 | 0.6 | Short | DI | Omnivore | 2.99 | 28.6 |
| *Secutor ruconius* | compressiform | superior | carangiform | 6.1 | 0.8 | Short | DI | Planktivore | 2.71 | 28.1 |
| *Setipinna tenuifilis* | compressiform | terminal | subcarangiform | 14.1 | 1.7 | Medium | DI | Planktivore | 3.60 | 28.4 |
| *Siganus fuscescens* | compressiform | sub-terminal/inferior | carangiform | 15.0 | 0.9 | Short | MM | Planktivore | 2.03 | 27.9 |
| *Sillago sihama* | anguilliform | terminal | subcarangiform | 15.6 | 1.1 | Short | DI | Zoobenthivore | 3.33 | 28.5 |
| *Solea ovata* | compressiform_flat | sub-terminal/inferior | anguilliform | 7.0 | 1.1 | Short | MS | Zoobenthivore | 3.50 | 28.3 |
| *Stolephorus commersonnii* | fusiform | sub-terminal/inferior | subcarangiform | 7.4 | 0.9 | Short | DI | Planktivore | 3.05 | 28.3 |
| *Takifugu ocellatus* | anguilliform | terminal | tetraodontiform | 10.0 | 0.7 | Short | DI | Miscellaneous | 3.25 | 24.7 |
| *Takifugu xanthopterus* | anguilliform | terminal | tetraodontiform | 29.0 | 2.2 | Medium | MM | Miscellaneous | 3.37 | 20.7 |
| *Terapon jarbua* | fusiform | terminal | subcarangiform | 20.8 | 3.1 | Long | DI | Omnivore | 3.93 | 23.7 |
| *Terapon theraps* | fusiform | terminal | subcarangiform | 19.8 | 1.2 | Short | MM | Zoobenthivore | 3.49 | 28.5 |
| *Thryssa kammalensis* | compressiform | sub-terminal/inferior | subcarangiform | 8.9 | 2.4 | Medium | MM | Planktivore | 3.37 | 29.0 |
| *Thryssa setirostris* | compressiform | sub-terminal/inferior | subcarangiform | 11.8 | 1.9 | Medium | MM | Planktivore | 3.32 | 28.5 |
| *Thryssa vitrirostris* | compressiform | sub-terminal/inferior | subcarangiform | 15.5 | 1.3 | Medium | MM | Planktivore | 3.39 | 27.4 |
| *Trachicephalus uranoscopus* | anguilliform | superior | subcarangiform | 7.0 | 1.4 | Short | MM | Piscivore | 3.62 | 28.6 |
| *Tridentiger barbatus* | anguilliform | terminal | diodontiform | 7.3 | 1.5 | Medium | ES | Zoobenthivore | 3.37 | 27.8 |
| *Trypauchen vagina* | anguilliform | terminal | diodontiform | 14.1 | 2.2 | Medium | DI | Omnivore | 3.50 | 28.2 |
| *Upeneus japonicus* | fusiform | sub-terminal/inferior | carangiform | 9.8 | 1.2 | Short | MS | Zoobenthivore | 3.58 | 19.2 |
| *Upeneus sulphureus* | fusiform | sub-terminal/inferior | carangiform | 13.4 | 0.8 | Short | MM | Zoobenthivore | 3.08 | 28.0 |
| *Uroconger lepturus* | anguilliform_eel | terminal | anguilliform | 30.0 | 2.1 | Medium | MS | Zoobenthivore | 3.50 | 16.0 |
| *Zebrias zebra* | compressiform_flat | terminal | anguilliform | 16.3 | 2.3 | Medium | MS | Zoobenthivore | 3.50 | 28.3 |

Note: EUFG: ES (Estuarine species); DI (Diadromous); MM (Marine migrant); MS (Marine straggler).

Life span: Long (>10 years); Medium (5–10 years); Short (<5 years).

Table S3. Fish species diversity and functional diversity indices among different sites in the Pearl River Estuary.

| Sites | Total species  (S) | Shannon  (H') | Gini-Simpson  (1-λ) | Pielou's evenness  (J') | Margalef  (d) | FRic | FEve | FDiv | FDis | RaoQ |
| --- | --- | --- | --- | --- | --- | --- | --- | --- | --- | --- |
| S1 | 5 | 1.35 | 0.70 | 0.84 | 1.36 | 0.06 | 0.83 | 0.87 | 0.29 | 0.09 |
| S2 | 4 | 1.33 | 0.72 | 0.96 | 1.67 | 0.03 | 0.86 | 0.88 | 0.25 | 0.07 |
| S3 | 23 | 2.48 | 0.87 | 0.79 | 4.51 | 0.20 | 0.74 | 0.73 | 0.33 | 0.11 |
| S4 | 18 | 2.00 | 0.80 | 0.69 | 3.09 | 0.21 | 0.75 | 0.75 | 0.32 | 0.11 |
| S5 | 10 | 2.13 | 0.86 | 0.92 | 3.06 | 0.15 | 0.87 | 0.77 | 0.33 | 0.11 |
| S6 | 21 | 2.33 | 0.86 | 0.76 | 3.58 | 0.19 | 0.75 | 0.73 | 0.32 | 0.10 |
| S7 | 33 | 2.71 | 0.90 | 0.77 | 5.99 | 0.30 | 0.73 | 0.77 | 0.33 | 0.12 |
| S8 | 4 | 1.34 | 0.73 | 0.97 | 1.08 | 0.03 | 0.86 | 0.72 | 0.25 | 0.08 |
| S9 | 15 | 1.84 | 0.78 | 0.68 | 3.01 | 0.13 | 0.75 | 0.80 | 0.30 | 0.10 |
| S10 | 18 | 1.74 | 0.71 | 0.60 | 3.02 | 0.18 | 0.75 | 0.76 | 0.30 | 0.09 |
| S11 | 13 | 1.74 | 0.68 | 0.68 | 2.80 | 0.13 | 0.85 | 0.82 | 0.31 | 0.10 |
| S12 | 9 | 1.35 | 0.65 | 0.61 | 1.94 | 0.17 | 0.78 | 0.74 | 0.30 | 0.10 |
| S13 | 35 | 2.88 | 0.91 | 0.81 | 6.35 | 0.31 | 0.80 | 0.79 | 0.32 | 0.11 |
| S14 | 15 | 1.72 | 0.67 | 0.64 | 3.22 | 0.22 | 0.77 | 0.82 | 0.32 | 0.11 |
| S15 | 3 | 1.04 | 0.63 | 0.95 | 1.44 | 0.05 | 0.90 | 0.69 | 0.28 | 0.08 |
| S16 | 22 | 0.95 | 0.36 | 0.31 | 3.10 | 0.18 | 0.75 | 0.66 | 0.33 | 0.11 |
| S17 | 26 | 2.57 | 0.86 | 0.79 | 4.91 | 0.31 | 0.80 | 0.77 | 0.33 | 0.11 |
| S18 | 20 | 1.72 | 0.65 | 0.57 | 3.35 | 0.25 | 0.82 | 0.75 | 0.32 | 0.11 |
| S19 | 0 | 0.00 | NA | NA | NA | NA | NA | NA | NA | NA |
| S20 | 5 | 0.16 | 0.06 | 0.10 | 0.57 | 0.04 | 0.73 | 0.90 | 0.23 | 0.06 |
| S21 | 19 | 1.37 | 0.57 | 0.46 | 2.90 | 0.20 | 0.84 | 0.63 | 0.32 | 0.11 |
| S22 | 8 | 1.25 | 0.59 | 0.60 | 1.66 | 0.06 | 0.75 | 0.76 | 0.29 | 0.09 |
| S23 | 29 | 2.79 | 0.91 | 0.83 | 5.68 | 0.30 | 0.80 | 0.72 | 0.33 | 0.11 |
| S24 | 10 | 2.00 | 0.83 | 0.87 | 2.76 | 0.20 | 0.85 | 0.91 | 0.33 | 0.11 |
